# Reimagining Freire: beyond human relations

**DOI:** 10.1007/s11422-023-10154-7

**Published:** 2023-03-17

**Authors:** Naya Jones

**Affiliations:** Department of Sociology and Global and Community Health Program, University of California Santa Cruz, 1156 High St, Santa Cruz, CA 95064 USA

**Keywords:** Relational, Freire, Black ecologies, Critical pedagogy, Environmental education

## Abstract

By bridging critical pedagogy and environmental praxis, the contributions in this forum build on Freire’s legacy while stretching his work. As the authors attend to more-than-human life, they theorize and enact relational ways of knowing. Through participatory and multisensory pedagogies, they counter dichotomies between nonhuman and human nature, student and teacher. In this response, I consider how this (re)centering of more-than-human relations expands—and counters—Freire’s thinking, including how he articulates humanization as a primary, liberatory aim of teaching and (un)learning. Along with insights from Black geographies and Black feminist ecologies, bell hooks guides my response. hooks critiqued and engaged with Freire’s work with radical care, with space for complexity and accountability. This way of reading feels particularly suited to this forum, as the authors reimagine Freire’s contributions to critical (environmental) pedagogy for the twenty-first century and beyond.

As I read this forum, I kept thinking of bell hooks’ reflections on Freire’s work. Really, thinking came second. First came a vision of hooks and Freire having tea or a meal together. The vision looks like a photograph: these two ancestors of critical pedagogy, having a conversation in the afterlife or in some other dimension where they meet again.

hooks recently passed away in December 2021, while Freire passed away in 1997. We know from hooks’ interviews that she treasured Freire’s work. We know *Pedagogy of the Oppressed* (1970) influenced her as a learner and teacher. In *Teaching to Transgress* (1994), she reflects on how Freire’s work resonated with her as an educator from “a rural southern [American] black experience” (46). She writes this reflection as a dialogue with herself, using her birth name Gloria Watkins and her chosen name of bell hooks. By doing so, she practices dialogic pedagogy on the page. And she reflects on how, when she came upon Freire’s work, she was seeking language for understanding and theorizing injustice. hooks describes Freire as part of her intellectual lineage, and this includes critiques of his work. hooks was not alone in her feminist critique of Freire’s language in books such as *Pedagogy of the Oppressed*, where he consistently refers to “he” or “Man” as the catalyst for critical consciousness. She raised this critique publicly with Freire, at an event where he was a speaker. When he responded, he practiced the pedagogy he advocated; he listened and stated a commitment. hooks recalls, “And he spoke then about making more of a public effort to speak and write on these issues—this has been evident in his later work” (56). In *Pedagogy of Hope* (1992), for example, Freire addresses feminist criticisms of his earlier work directly and at length. This moment further inspires that vision of hooks and Freire sharing a meal: there is a sense of meaningful dialogue.

The pieces in this forum both build on Freire’s work and stretch his work, and hooks models how to be with the complexity of his legacy. In their articles, the authors emphasize process, challenge the “banking” method of education, engage in learning beyond the conventional classroom, and challenge dichotomies between “student” and “teacher.” They contextualize learning within broader power dynamics and histories; they articulate relationships between theory and practice. As they do so, they offer practices fellow educator-activists may take forward. Following Freire’s emphasis on communication, these articles attend to communication as not only essential for learning but as a site of learning. Their praxis is deeply relational, in the sense of fostering and centering relationship. At the same time—and here is where hooks’ way of critique becomes important—the forum authors stretch Freire’s critical pedagogy beyond human relations. As they reimagine critical environmental education, they acknowledge and theorize how human and ecological relations entwine, how they are mutual. As they analyze “collective becomings” through an art and permaculture cooperative in Argentina (Martín et al.) and a youth naturalist program in the United States (Hecht), this relationality ranges from situating human relations within nonhuman nature, to tracing human–environment relations, to centering the agency of nonhuman nature, such as animals, plants, and water. The authors emphasize plural knowledges as they (re)center Indigenous, local, and multisensory ways of knowing alongside Western science. While the terms “nonhuman nature” and “more-than-human” are less-than-optimal and still do not quite capture relational ways of knowing, I use them throughout this response to better draw out the relationships lifted up by the authors. They theorize and enact relational ways of knowing which assume the interconnectedness of nonhuman and human life, center relationship, and counter Western dichotomies between nonhuman/human nature, mind/body, and student/teacher, among others. As they do, they insist on engaging with more-than-human relations for collective and planetary thriving.

I begin by *Situating* this forum within the ever-evolving landscape of research on relationality in critical and environmental education. Rather than an exhaustive literature review, I underscore some of the works that this forum brings to mind. This brief review also reflects what I have been thinking and feeling through as an educator–scholar. In *Grounding*, I share more about my location at the crossroads of Black geographies, Black feminist ecologies, and embodied praxis. This interdisciplinary foundation shapes my response, while teaching about Black botanical geographies inspires my commitment to relational and reflexive pedagogies. From here, I revisit how Freire centers human relations, including key critiques about the anthropocentrism of his work. When I turn to the pieces in this forum, I propose that their attention to more-than-human relations involves much more than illuminating yet another “set” of relationships. The authors inspire questions: By bridging Freire’s insights on critical pedagogy with wide-ranging environmental theory and practice, how do the authors extend (and even counter) Freire’s work? How do they enact relationality, not only as a concept, but as living practice? How does doing so disrupt underlying assumptions of Western science? And, why is this reimagining critical now?

In this response, I engage with these questions and raise others. hooks especially guides how I critique Freire’s work. As a fellow Black educator based in the United States context, I share hooks’ appreciation of Freire’s work. I mutually appreciate how Freire offers language for understanding and dismantling power dynamics, for harnessing collective power. Furthermore, in the spirit of Black feminism, hooks considers her lived experiences with race, gender, class and other structural identities integral to her intellectual autobiography. My interdisciplinary location is inspired by living, studying “old ways,” and community organizing as a Black and Blaxicana educator-researcher. I am practicing awareness of both the privileges and oppressions I bring to this response as a Black scholar based in the Global North. By reading the forum articles in conversation, and by being in conversation with them, I seek to practice the dialogue hooks and Freire lift up. May I bring openness and a willingness to learn.

## Situating

These case studies complement and extend work on *relationality* in critical science and critical environmental education: When Rocio Martín et al. emphasize plural knowledges and the necessity of engaging Indigenous and Western scientific thought, they bring to mind Megan Bang and Marin’s (2015) critical analysis of what putting Indigenous and Western science in conversation requires, specifically in terms of reframing nature–culture, aka nature–human, relations. They evoke calls to engage with Indigenous science, both in terms of *conceptualizing* relationality and *practicing* relationality, reciprocity, and responsibility; in other words, relationality extends beyond analysis into everyday praxis (Bang, Marin and Medin 2018). By grappling with multiple knowledges, Martín et al. reflect a recurring thread throughout the forum and in the broader field of critical environmental education, as scholars underscore Indigenous ways of knowing as relational—and the necessity of centering these onto-epistemologies with care. Nxumalo, Nayak and Tuck (2022, 98) argue that this centering must attend not only to principles but to the contemporary Land Back movement and the impact of climate change on Indigenous lives; pedagogy grounded in Indigenous ways of knowing enacts “radical relationality” which “take[s] seriously the knowledge-making capabilities of the more-than-human world” (also visit Nxumalo and Montes 2021).

When Hecht practices critical self-reflection about an urban naturalist program in Philadelphia, she disrupts Western scientific dichotomies between human and nonhuman nature, teacher and student. Her attention to how community is cultivated through difference and (in) justice, among human and nonhuman life, recalls how Bazzul and Tolbert (2018) theorize the pedagogical commons: a space where conventional distinctions between the “social” and the “natural” are intentionally blurred. Drawing on assemblage theory, they emphasize how commons bring together human and nonhuman life. Read with Hecht, they expand on the precarity of the commons as well due to privatization and state interests; they further situate what Hecht describes as “learning refugia” in broader socio-political context. Taken together, the case studies invite us to witness relational pedagogies with intimate ties to place, while situating these within broader power dynamics.

## Grounding

This brief situating reflects the crossroads where I do “the work”. For research and pedagogy, I attend to how relational epistemologies have been carried forward and carried out through community-led Black botanical medicine. This involves attending to everyday relations with plants for medicine and as medicine; to relations with plants in expressive culture such as poetry and music; to ritual relations with plants and how plants connect with people; to the agency of plants themselves, not only as teachers or kin, but on their own terms. Without grounding in critical theory, this pedagogy risks perpetuating colonial legacies, among them the reductive “listing” of plants and practices without addressing power. Such cataloguing facilitated colonialism and genocide; this cataloguing undergirded hierarchical taxonomies of people, animals, and plants, laying the groundwork for scientific racism and its legacies (Smith 2012). Building on Freire (1970), one way students and I counter these legacies is by practicing reflexivity. Rather than studying Black botany as a subject *outside* of ourselves, we consider our social locations, roles, and relationship to Black botany as living practice and philosophy with implications for the planet—people, plants, animals, earth. But reflexivity does not necessarily disrupt human/nature dichotomies.

To further disrupt dichotomies, I turn to Black geographies and Black ecologies. Black geographies underscore the ways in which space and place constitute power dynamics and legacies of colonialism while lifting up Black world-making (Hawthorne 2019). As a related field, Black ecologies often builds on and with Black geographies, while accenting how Black world-making has always involved, acknowledged, and cultivated more-than-human relations. Through this frame, contemporary socio-ecological relations reflect legacies of colonialism and white supremacy. The afterlives of enslavement, Bruno (2022) raises, are also biophysical, with material ecological impacts for human and more-than-human life. As legacies of oppression persist, so do ecological legacies of resistance, refusal, and thriving (Hosbey and Roane 2018). In order to understand these entwined legacies (and counter antiblackness), interventions from Black ecologies and Black geographies are thinking relationally, with attention to how Black and Indigenous epistemologies, histories, and movements involve each other. Nxumalo (2018) considers how placing Black and Indigenous feminisms in conversation, for example, can unsettle child-centered pedagogies, counter the erasure of Indigenous onto-epistemologies, and center the impact of climate change on/with Indigenous communities. Along with these interventions, I am interested in how thinking and feeling relationally dismantles destructive human/nature dichotomies and undermines counterproductive dichotomies constructed *between* epistemologies—dichotomies such as Western science/Indigenous science, or dichotomies constructed between the epistemologies of historically-oppressed communities. Voeks and Rashford (2013) outline how Indigenous knowledge has been romanticized and assumed, while Black diaspora plant knowledge—along with diasporic and migrant (ethno)botany in general—is too often dismissed. Reading relationally surfaces points of connection and disjuncture across “othered”, subjugated epistemologies.

I am also thinking with Davis, Moulton, Van Zant and Williams (2019) as I consider how to teach relationality amid climate change, COVID-19 and its legacies, and more. Echoing widespread critiques of the Anthropocene as a concept, Davis et al. underscore the need to displace the Anthropocene narrative, in no small part because its universal “we” bypasses historical and contemporary power dynamics. They instead reframe the Plantationocene. They agree with how Haraway, Ishikawa, Gilbert, Olwig, Tsing and Bubandt (2015) discuss the Plantationocene in some respects: they concur with centering the plantation economy as catalytic for climate change rather than the Industrial Revolution, with approaching the plantation as a site of multispecies relationship, and with the necessity of “making kin” across species. However, Davis, Moulton, Van Sant and Williams (2019) emphasize the human labor and racial violence integral to plantation economies. Expanding on Wynter (1971), they underscore the “plot” as both a literal site and an analytic: a literal site of resistance, sustenance, and multispecies kinship with plants, animals, and other life among maroons as well as enslaved Africans and their descendants, and an analytic for considering “a relational mode of being” (2019, 8). Significantly, this relational mode extends to metaphysical relations such as ancestors or spirit/Spirit. As Harris and Roane (2022) point out, the metaphysical is all too often pruned from accounts and studies of Black life. All too often, these dimensions are deeply misrepresented in academic and public discourse, though they may be central to everyday lives—and although the metaphysical materializes through everyday relations with nonhuman nature, from plants to rivers  (Gumbs 2018; Henery 2020, 2021; Jones 2021).

This expansive understanding of more-than-human relations undergirds my response, along with a desire to teach in a more deeply relational way. In the context of climate change education, Nxumalo, Nayak, and Tuck (2022, 103) raise how “climate change education needs to be more than a cognitive ‘learning about’; it requires embodied and affective immersion in and ethical commitments towards building alternative worlds and relations”. Beyond teaching about relational ways of knowing, I seek to put them into practice. This past Spring in a Black Botanical Medicine in the Americas course, we practiced learning *from* and *with* plants through embodied exercises, critical self-reflexivity, and group commitments. Students felt and thought through course concepts in different forms of their choosing, from short essays to collage and poetry. While this class drew on some of my past work on critical and embodied pedagogy (Jones 2019; Thomas and Jones 2019), we were also practicing and learning together in a newly remote context due to COVID-19. At the end of the quarter, I felt as if we had touched on relational modes of being. But I also wondered, how much more immersive could this learning be, whether remote or in person? How to put these more-than-human relations into practice, and how to articulate them in/as critical pedagogy? As the forum authors address similar questions, they stretch and counter Freire’s articulation of critical pedagogy in significant ways. To understand this significance requires a revisiting Freire’s take on human/nonhuman relations.

## Revisiting Freire and (human) relations

*Pedagogy of the Oppressed*, Freire’s most well-known text, centers on human relations: from power relations between and among people, within countries, and across place; to dialogue and listening as the foundation of critical pedagogy; to practicing hope and love with fellow humans. *Conscientização*, as both an intention and guiding concept in Freire’s work, requires active engagement with all of these in order to recognize oppression and catalyze transformation. While power relations constitute context, dialogue and listening allow for reimagining human relations. Central to this reimagining, is the recognition of the oppressed in their “fuller humanity,” first and foremost by themselves (but also by the oppressor). In *Pedagogy of the Oppressed*, he argues, “To surmount the situation of oppression, people must first critically recognize its causes, so that through transforming action they can create a new situation, one which makes possible the pursuit of a fuller humanity” (47). Dialogue is necessary, he proposes, for practicing fuller humanity, and the capacity for dialogue, the capacity to transform the world, distinguishes the *oppressed* from *things*. Freire holds, “In order to regain their humanity they must cease to be things and fight as men and women,” and he continues, “They cannot enter the struggle as objects in order later to become human beings” (68). Freire understands oppression as dehumanizing, for both the oppressed and the oppressor: liberation and humanization entwine (more on this later). There is, of course, much more nuance in *Pedagogy of the Oppressed*, and in Freire’s broader body of work, but these examples point to how Freire centers human relations and humanization.

When Freire discusses nonhuman life such as animals or plants, he typically does so to differentiate what *becoming human* means. Here is where provocative critiques and debates about his analysis of nonhuman nature arise. For instance, in *Pedagogy of the Oppressed*, *becoming* is the purview of humans. Freire (2005, 97) writes, “Indeed, in contrast to other animals who are unfinished, but not historical, people know themselves to be unfinished; they are aware of their incompletion. In this incompletion and this awareness lie the very roots of education as an exclusively human manifestation.” In *Pedagogy of Hope* (2005, 90), he argues, “Throughout history, we men and women become special animals indeed, then. We invent the opportunity of setting ourselves free to the extent that we become able to perceive as unconcluded, limited, conditioned, historical beings. Especially, we invent the opportunity of setting ourselves free by perceiving, as well, that the sheer perception of inconclusion, limitation, opportunity, is not enough.” He makes similar observations of plants, which warrants particular attention for this forum because the authors teach through gardens, farms, and urban parks: botanical life is central to their critical pedagogy. Freire observes: “The transformation occurring in a seed which under favorable conditions germinates and sprouts, is not development. In the same way, the transformation of an animal is not development. The transformations of seeds and animals are determined by the species to which they belong; and they occur in a time which does not belong to them, for time belongs to humankind” (2005, 161). Here, plants and animals lack agency as well as the capacity to transform and exist outside of time, or perhaps outside of time in a sense that *matters*.

Misiaszek and Torres (2019) argue that Freire centers humans to emphasize responsibility rather than superiority. They acknowledge the emphasis on human relations in Freire’s work, and as Hecht cites in this forum, they describe ecopedagogy as “the missing chapter of *Pedagogy of the Oppressed*.” The missing chapter Misiaszek and Torres envision, are not a critique of anthropocentrism in Freire’s work. Rather, they imagine this chapter, envisioning what Freire might have written about oppression of the environment. As they construct this chapter based on his body of work, they approach ecopedagogy as teaching focused on “connections between human acts of environmental ills and social conflict (socio-environmental issues) for praxis to end oppressions”; they emphasize how this is one way to understand ecopedagogy, inspired by Freire’s environmental thought (465). Following Freire, this definition points to human acts and their impacts, alongside the human capacity to transform those acts. They carry this argument forward when they differentiate *the World*, defined as human interactions, from *the Earth*, or nonhuman life. Again, this differentiation extends Freire’s arguments: “All organisms utilize their environments; however, only humans reflect upon their actions upon Earth—the environment, other animals, and each other (i.e., the World)” (472). For Misiaszek and Torres (2019), Freire differentiates humans from nonhuman life, but does not render humans superior to nonhuman life.

Among critiques of Freire’s work, Corman (2011) outlines three themes regarding nonhuman animals in *Pedagogy of the Oppressed*: animals as noncommunicative, without agency, and without history or culture. Corman notes how this representation stems from assumptions of human superiority as well as from Freire’s emphasis on dialogue as the “antidote to oppression” (34). This critique builds, in part, on Bell and Russell (2000), whose critique underscores Freire’s characterization of animals as “impossible subjects”; they further address the ways in which the emphasis on dialogue and language is used to render humans distinct from nonhuman life, such that, “language undermines our embodied sense of interdependence with a more-than human-world” (p. 193). These critiques mutually raise how Freire makes assertions about animal (and plant) life as if they are commonsense: underlying dichotomies within Western scientific thought bolster his argument. At stake, these authors argue, is the perpetuation of human/nonhuman dichotomies which underlie the mass mistreatment and killing of animals (Corman 2011). Bell and Russell (2000) point to how the reification of humans as separate and superior from nonhuman life perpetuates intersecting environmental crises, while relying on a dichotomy used to justify oppression of human groups considered “closer to nature” (p. 193).

Misiaszek and Torres point to critics’ limited engagement with Freire’s broader scope of work. They emphasize the need to engage with his full body of work, to approach his work as *becoming*. To be sure, the critiques noted here do focus on the *Pedagogy of the Oppressed*. But what are we to make of the fact that this is Freire’s most influential and wide-reaching work? Or, of how he makes similar arguments elsewhere, for example as I mentioned in *Pedagogy of Hope*? Still other questions (and openings) arise when reading interventions from Black Studies alongside Freire’s work. For example, Jackson (2020) unsettles the category of “the human” and “the animal” in ways that challenge the aim and scope of critical (environmental) pedagogy. The critiques noted above acknowledge how these categories are deeply racialized and gendered. Through the lens of Black cultural production, Jackson further underscores how humanization is not necessarily humanizing, partly because blackness continues to be characterized as animalistic. Engaging the question of humanization through blackness, she points out, disrupts the notion of a distinct (and rigid) human/nonhuman animal binary. Furthermore, and of particular interest here, an emphasis on humanization continues to exclude “life writ large” beyond humans alone, while deconstructing “the human” reimagines relations (Jackson 2020, 34). Jackson offers an expansive definition of relationality, when she considers how Black cultural producers cultivate “nonbinaristic models of human–animal relations” and advance “theories of trans-species interdependency” (p. 18). In addition to further situating humanization within legacies of colonialism, creativity, and relational ways of knowing, Jackson brings me back to the centrality of “becoming human” in Freire’s work. Throughout *Pedagogy of the Oppressed* and later works, Freire conceptualizes humanization *as* liberation. He writes, “[Humanization] is thwarted by injustice, exploitation, oppression, and the violence of the oppressors; it is affirmed by the yearning of the oppressed for freedom and justice, and by their struggle to recover their lost humanity” (2012, 44). He acknowledges the implications of oppression, including how oppression dehumanizes everyone. However, humanization and dehumanization occupy a binary here. The oppressed and the oppressors, are either humanized or dehumanized; Freire assumes humanization universally attainable. In a sense this speaks to his politics of radical hope, *and* this universality risks minimizing racial, sexual, and other hierarchies. Put plainly, if humanization is a dialectical process that requires agency and recognition, does everyone really have the ability to be recognized as human? What of how the category of “human” relies on colonial legacies?

By interrogating the category of “the human”, Jackson and work by scholars such as Sylvia Wynter and Katherine McKittrick challenge humanization as an emancipatory aim, while (re)turning to more-than-human relations. In terms of critical pedagogy, these interventions open up the aims and scope of critical (environmental) education. Although the arguments touched upon in this section debate the position of the human in Freire’s work, each presses for careful consideration of more-than-human relations for collective and planetary thriving.

## Reimagining relational praxis

Which brings me to the pieces in this forum. By bridging theory and practice, the authors in this forum not only assume relationality in terms of interconnectedness, implication, and involvement, but they seek to cultivate and practice these. In the case of Reciclando Utopias (Recycling Utopias), relations again encompass humans as well as nonhuman nature. Martín et al. describe the cooperative as a “learning context,” grounded in art and permaculture in Central Argentina for almost 10 years. Through practices such as design workshops and theatre, eco-construction and crafting sustainable products, Reciclando Utopias cultivates relations among community members, and between people and nonhuman nature. While one aim of the cooperative has always been to create meaningful work and provide jobs, Reciclando Utopias further creates opportunities to learn and reimagine relations. Here relations are explicitly social and ecological; and in some aspects, these become one in the same. For example, building on the work of Mollison and Holmgren, among others, the cooperative understands permaculture as “a holistic design system based on direct observation of nature, learning from ancestral knowledge and findings of modern science” (Martín et al., this issue). All coop activities emphasize “permaculture in everyday life”, such that permaculture constitutes social and ecological praxis: praxis that attends to cooperative, democratic social relations among humans, always in relationship with nonhuman nature and the planet. When witnessing the cooperative, the word *generative* comes to mind.

By creating spaces of “encounter” and workshops at the intersection of arts and permaculture, the APC attends to human relations in a Freirean sense. For example, some of the encounters are designed for the cooperative members, to promote learning among members and community-building, while others are for external participants. In doing so, the cooperative offers multiple opportunities for collaborative reflection. Furthermore, the coop considers how learning and teaching can be simultaneous, reflecting Freire’s point in *Pedagogy of the Oppressed*. In this case, however, nature is also a teacher. The authors include testimonies from APC members. I keep returning to how the APC president describes the water cycle and the everyday washing of hands: “You don’t do anything to complete the water cycle. As soon as you understand that water is a living being you start to think differently. You are not washing your hand, they are washing your hand” (Martín et al., this issue). This testimony points to how members of the cooperative expand *relations*. Water is not a resource to *manage* here; water is agentic and alive. Water teaches.

I keep returning to these words. They move me; they evoke Freire’s attention to love and hope as not only emotions but as catalysts for transformation. Throughout, this case study lifts up how love, hope, and more might be cultivated and expressed through learning. In doing so, the authors open up questions about art and aesthetics in critical environmental education. How is critical environmental education not only about which relations are expressed, but about *how* they are expressed? How does the art and permaculture cooperative nuance human relations, by engaging mind, body, and spirit (although I wonder what language the cooperative members themselves might use here)? How does this attention to expression extend to “writing up” research? For instance, Martín et al. begin with a poem on “recycling utopias,” the inspiration for the cooperative. Through the cooperative, art in the form of poetry, theatre, and more are mobilized as a way to both *express* social–ecological relations and *learn* about them. I also wonder how expressive modes of learning and teaching facilitate paradigm shifts. Building on permaculture, bridging “ancestral knowledge *and* findings in modern science” (italics mine), involves tensions. The authors point to these tensions when they emphasize holding multiple knowledges, building on de Sousa Santos’ theory of an ecology of knowledges. What power dynamics must be acknowledged and named, to hold these multiple knowledges? How does understanding water as agentic, relate to Indigenous and Traditional ways of knowing that have already been practiced locally or globally? And, how/does permaculture make its engagement with Indigenous Knowledge in this context?

Similar engagements with relations between human and nonhuman life appear in Hecht’s piece, as she reflects on her experience teaching and learning with the Young Naturalists Program in the United States, as both a co-founder and educator. Grounded in ecopedagogy, this paid summer internship for youth addresses both social and environmental issues, and Hecht considers how interactions within the program are situated within intersecting broader power relations such as race/racism, class/classism, gender/sexism, among others. She attends to human and social relations by addressing power dynamics and interactions. Furthermore, her vivid descriptions of human interaction encompass language, emotions, and nonverbal modes of communication. Hecht reminds us of how communication can take many forms. And, by writing autoethnographically, Hecht practices reflexivity: *how* her autoethnographic reflection is written is part of ecopedagogy *as* praxis. Building on the work of Katherine McKittrick (2021), she explicitly approaches this reflection as storytelling. On the page, this looks like storytelling and analysis, interspersed with still other paragraphs where Hecht catalogues observations about plants, people, and animals and the relationships between them. These mini-catalogues remind me of entries from (ethno)botanical field notes, although in this case these might be considered counter-notes that seek to disrupt colonial legacies through intersectional analysis of power relations, critique of environmental destruction, and more.

Her observations especially reflect on *community*, and she expands on the concept of *learning refugia* as a way to hold human diversity and biodiversity (nonhuman life) together in pedagogical context. As a learning context, the Young Naturalists Program fosters dialogic learning, in part by intentionally involving youth from different racialized, class, geographic, and other backgrounds. Hecht observes how dialogic learning emerges without specific prompts, but instead in the course of collaborative work as the group “cut down vines choking young trees; hauled logs to build erosion and deer protection structures; learned names of birds and trees and insects; held moths gently in our hands; talked and listened to each other, a group of people that might never connect in other circumstances; discovered that these people were our people.” As Hecht suggests, and as Freire emphasizes, this kind of emergent dialogic learning is not guaranteed. Intentional human relationships are fostered. As I witnessed Hecht’s reflection, I wondered what scaffolding supported the learning? Would Hecht consider practices like community agreements or otherwise, as scaffolds for the space? And what did/do these look like, as the youth learn about nature with/in nature?

In fact, I appreciate how Hecht specifies *nature*, including the common and binomial names for plants, insects, and animals. As she discusses cultural and social diversity among humans, she lifts up diversity among other forms of life. But rather than uphold a human/nature binary, I think Hecht grapples with humans *as* nature as well, as she is “working to reveal the connections between biological and cultural diversity and the ways in which the loss of one is intimately tied to the other.” Relations again take multiple forms here, as Hecht considers how human relations within the program, unfold amid intricate, ecological relations that do not require human intervention or awareness. She writes, “microscopic fungal organisms [Mycorrhizae] connect trees to one another, connect trees to other flora. Each part not only relies on other parts of the system, they are part of one another, making up the whole of the thing we call forest. There are no individuals here. The value of diversity is for the collective, for the community” (Hecht, this issue). As she centers Mycorrhizae, Hecht moves beyond nature-as-metaphor; her observations are about more than equating the importance of biodiversity to human diversity, but about how these are always in relationship and involve each other. Meanwhile the words “what we call the forest” opens up space for wonder. What are names for forests? What might forests call themselves? Hecht inspires this wonder, partly because she emphasizes curiosity and inquiry, but also because she centers the lives of Mycorrhizae.

## Openings

I started with a vision of hooks and Freire in conversation. Returning there, I wonder what hooks and Freire would say to each other about relationality. Throughout her life, hooks described being moved by nonhuman nature as a child growing up in the agrarian South. As an adult, she drew upon the Buddhist teachings of Thich Nhat Hanh; on more than one occasion she references the relational concept of *interbeing*, the interconnectedness of all life and the aliveness of nonhuman nature. Maybe she would share this with Freire, along with the personal stories she shares throughout her body of work. In one short essay, she invites the reader to, “Stand on solid ground and be a true witness” (2008, 71). Her invitation as manifold. For Black (American) readers, I hear a call to remember more-than-human relations, to refuse white supremacy and capitalism by remembering longstanding Black ecological knowledge. For all readers, hooks means *solid ground* in a literal and figurative sense. Literally: stand on and with the ground. Remember the earth and nonhuman life feel, know, and sense—whether we are witnessing or not. Figuratively: keep rooted in legacies. Being *moved* by nonhuman nature is not outside of social, political, and ecological realities, as the forum authors point out. This point feels particularly meaningful, because Freire also engages with nonhuman nature outside of comparison with humans. For instance, he describes moments of communion with nonhuman nature and finding beauty in his wife’s garden (Freire 1992).

Still, the pieces in this forum remind us that disruption of the human/nonhuman divide, must go beyond appreciation. Collective becoming requires a more-than-human, relational, and connected pedagogy. Ananda Marin notes: “The first leap [in more-than-human relations] is always an affective leap—it feels so good to be outside, it feels so good to be listening to birds and watching the waves. All of that is important but it doesn’t help us to answer these larger questions of how do we understand territory, how do we understand migration, how do we understand our responsibilities to one another, how do we understand our stories, and how do we live our stories. So, the second leap is socio-political and temporal” (in Bang et al. 2022, 158). Freire addresses human relations at length. relational in the sense of upholding communication, dialogue, and mutual recognition of humanity. However, Freire does not make that leap to a more expansive relationality, one that engages more-than-human life such as animals and plants, or nonhuman nature such as water, as agentic and knowledge-producing. Moreover, interrogations of “the human” raise questions about the emancipatory possibilities of humanization.

Similar to how Misiaszek and Torres (2019), these case studies imagine what critical environmental pedagogy grounded in Freire might look like. They bridge Freirean critical pedagogy with wide-ranging environmental theory and practice. While the authors celebrate Freire’s contributions, their contributions do more than extend his theories. By engaging with more-than-human life, they raise where his work diverges from (and even opposes) critical environmental pedagogy. They surface tensions and gaps; they open up space for creative dialogue. hooks’ way of critique has guided me here, because she approaches critique with such radical care. I imagine hooks pressing Freire to address critiques of his human-centered analysis, as he addressed feminist critiques: directly and at length. I imagine her pressing *and* still finding meaning in his work. She would repeat: “I came to Freire thirsty, dying of thirst (in that way that the colonized, marginalized subject who is still unsure of how to break the hold of the status quo, who longs for change, is needy, is thirsty), and I found in his work (and the work of Malcolm X, Fanon, etc.) a way to quench that thirst. To have work that promotes one’s liberation is such a powerful gift that it does not matter so much if the gift is flawed” (1994, 50). hooks is not denying accountability (remember how she held Freire accountable). With accountability, she names a way forward. Corrections can be made. Missing chapters can—and must—be written. In the same spirit, this forum reimagines Freire for the twenty-first century and beyond.
